# Multimorbidity as a predictor of health service utilization in primary care: a registry-based study of the Catalan population

**DOI:** 10.1186/s12875-020-01104-1

**Published:** 2020-02-17

**Authors:** D. Monterde, E. Vela, M. Clèries, L. Garcia-Eroles, J. Roca, P. Pérez-Sust

**Affiliations:** 1 Sistemes d’Informació, Institut Català de la Salut, Barcelona, Catalonia Spain; 2 Unitat d’informació i Coneixement, Servei Català de la Salut, Barcelona, Spain; 3 Gerència de Sistemes d’informació, Servei Català de la Salut, Barcelona, Spain; 4 Hospital Clinic de Barcelona, Institut d’Investigacions Biomèdiques August Pi i Sunyer (IDIBAPS), CIBERES, Universitat de Barcelona, Villarroel, 170, 08036 Barcelona, Spain; 5 Coordinació de les Tecnologies de la Informació i la Comunicació del Sistema de Salut. Generalitat de Catalunya, Barcelona, Spain

**Keywords:** Chronic care, Comorbidity, Primary care, Health service utilization, Risk assessment

## Abstract

**Background:**

Multimorbidity is highly relevant for both service commissioning and clinical decision-making. Optimization of variables assessing multimorbidity in order to enhance chronic care management is an unmet need. To this end, we have explored the contribution of multimorbidity to predict use of healthcare resources at community level by comparing the predictive power of four different multimorbidity measures.

**Methods:**

A population health study including all citizens ≥18 years (*n* = 6,102,595) living in Catalonia (ES) on 31 December 2014 was done using registry data. Primary care service utilization during 2015 was evaluated through four outcome variables: A) Frequent attendants, B) Home care users, C) Social worker users, and, D) Polypharmacy. Prediction of the four outcome variables (A to D) was carried out with and without multimorbidity assessment. We compared the contributions to model fitting of the following multimorbidity measures: i) Charlson index; ii) Number of chronic diseases; iii) Clinical Risk Groups (CRG); and iv) Adjusted Morbidity Groups (GMA).

**Results:**

The discrimination of the models (AUC) increased by including multimorbidity as covariate into the models, namely: A) Frequent attendants (0.771 vs 0.853), B) Home care users (0.862 vs 0.890), C) Social worker users (0.809 vs 0.872), and, D) Polypharmacy (0.835 vs 0.912). GMA showed the highest predictive power for all outcomes except for polypharmacy where it was slightly below than CRG.

**Conclusions:**

We confirmed that multimorbidity assessment enhanced prediction of use of healthcare resources at community level. The Catalan population-based risk assessment tool based on GMA presented the best combination of predictive power and applicability.

## Background

Multimorbidity, defined as the coexistence of two or more diseases in a given individual, is a common feature in chronic patients [1, 2] that increases with age [3] and is well recognized as one of the major burdens on health systems worldwide [4, 5]. Multimorbidity shows well established associations with both high use of healthcare resources and poor patient prognosis [6]. Regarding its impact at community level, multimorbidity shows positive correlations with number of outpatient visits [7], polypharmacy [8] and with patients’ frailty [9]. The latter being a strong modulator of the need for both homecare and social support services [10, 11].

Prevention and management of multimorbidity requires implementation of care coordination, which involves integration of health and social services, in order to face the challenges associated with the increasing prevalence of chronic disorders [12, 13]. In this regard, the efficient implementation of the chronic care model appears as the best way to ensure health value generation, equity and sustainability of health systems [14, 15].

It is acknowledged that appropriate assessment of multimorbidity [3, 7, 16] constitutes a core need in order to enhance service commissioning, as well as other health policy issues associated with large scale deployment of the practicalities of the chronic care model. To this end, the current research uses a population-health approach to compare the predictive role of four well-defined modalities of multimorbidity assessment on use of healthcare resources in Primary Care. Moreover, the current study is to provide an objective assessment of the new tool for population-based health risk assessment (GMA, Adjusted Morbidity Groups), developed in Catalonia and implemented in Spain since 2015, in terms of prediction of use of healthcare resources in primary care.

## Methods

### Data source and study population

Since 2011, the Catalan Health Department surveillance system (CHSS) collects detailed information on healthcare usage for the entire population of Catalonia (North-Eastern Spain, 7.5 million inhabitants) [17]. It includes information from hospitalization, primary care visits, emergency department visits, skilled nursing facilities, palliative care and the mental health services, information on pharmacy prescription and expenditure, and a registry on the billing record also encompassing outpatient visits to specialists, home hospitalization, medical transportation (urgent and non-urgent), ambulatory rehabilitation, respiratory therapies and dialysis.

The registry has an automated data validation system that checks the consistency of the data and identifies potential errors. Moreover, as this information is used for provider payment purposes, external audits are performed periodically to ensure the quality and reliability of the data. The CHSS is also used to elaborate, on a six-month basis, the regional population-based health risk assessment tool, known as GMA (Adjusted Morbidity Groups), which generates the health risk strata pyramid of the general population of Catalonia [18, 19].

For the purposes of the current study, all adult residents (≥18 years) in Catalonia on 31st December 2014 were included in the analysis. This yielded a final study population of 6,102,595 cases. The research was undertaken under the umbrella of the Nextcare project [20], approved by the Ethical Committee for Human Research at Hospital Clínic de Barcelona (HCB/2018/0805). We used retrospective de-identified data from administrative databases and, therefore, the need for informed consent was waived.

### Multimorbidity assessment

The study compared the predictive power of four different measures assessing multimorbidity: the Charlson index [21], number of chronic diseases, Clinical Risk Groups (CRG) [22], and GMA [18, 19, 23].

The Charlson index was included because it is the most broadly used parameter to assess multimorbidity [3]. This index was initially developed in hospitalized patients to estimate mortality prognosis based on age and the fixed weights of 20 specific disorders [21]. The current study used the 2007 updated version of the Charlson index [24] adapted to primary care, further refined in 2014 [25].

The number of chronic diseases was based on the Clinical Classifications Software (CCS) [26] and the Chronic Condition Indicator (CCI) [27] elaborated by the Healthcare Cost and Utilization Project (HCUP) of the Agency for Healthcare Research and Quality (AHRQ). The CCS aggregates all diagnosis codes into 262 mutually exclusive, clinically homogeneous categories; whereas the CCI allows to determine if a diagnosis is a chronic condition. The combination of CCS and CCI provides the number of chronic conditions for a given subject.

The study also included information provided by the Clinical Risk Groups (CRG), elaborated to predict total annual health costs for large patient groups [22]. CRG consist of mutually exclusive risk groups estimating past and future use of healthcare resources. It is of note that calculation of CRG required information on diagnosis across the Catalan health system during 2014, as well as data on pharmacological prescriptions during the same period. Only estimation of future use of resources was considered in the current study.

Finally, we assessed the role of the morbidity grouper developed in Catalonia (GMA) [18, 19]. GMA classifies the population into 31 mutually exclusive categories based on both multimorbidity and levels of patient complexity (see detailed information on the GMA’s algorithm and validation in Additional file 1: Figures S1–S3).

### Outcome variables

The outcome variables considered in the current study were: (A) *Frequent attenders in primary care*, defined by ≥12 visits to the primary care team irrespective of the professional (physician, nurse, physiotherapist, etc.) and the type of visit (primary care unit, home, remote) during the year 2015; *(B) Patients receiving home care support* either by the primary care team, emergency services or teams specialized in geriatric and palliative care during 2015; *(C) Patients receiving social support visits* defined as patients that performed visits to the community-based social care worker during 2015; and, *(D) Patients receiving polypharmacy*, defined by prescription of more than eight drugs during the year 2015. All the outcome variables were treated as dichotomous.

A sensitivity analysis carried out to determine the cut-off points for A) and D) showed similar results for percentiles 95 and 85. The later (P85) was used in the study. The analysis of patients receiving social support was limited to the primary care centres that had one social worker assigned to the staff (*n* = 4,776,005) due to the fact that in some geographical areas social support is directly linked to city council services and information was not available for the current analysis.

### Data analysis

The current study consisted of a prospective analysis of existing registry information at 31 December 2014 to calculate the four multimorbidity measures (Charlson index, number of chronic diseases, CRG and GMA) and the events occurring during the entire 2015 for the four outcomes variables (A to D) described above. Results are expressed as mean values, standard deviations and 95% confidence intervals.

Logistic regressions were carried using each outcome variable as the dependent variable. For each model, the following covariates were considered: (i) age, (ii) sex, and (ii) socioeconomic level, as well as all first order interactions among those covariates. Moreover, the individual contributions of the three multimorbidity measures to the performance of the resulting predictive models was assessed, with a log-likelihood ratio test, through its inclusion as a covariate in the regression analyses. Accordingly, the model with age, sex and socioeconomic status was the baseline model. Age was analysed as a categorical variable grouped in 5-year intervals except for the two extreme periods, 18–19 years and > 94 years. Socioeconomic level was calculated as average income of all the residents living in the primary care area and expressed as a categorical variable using five levels [28]. It is of note that multimorbidity measures were included in the predictive modelling as categorical variables to allow for possible non-linearity in the relationship between multimorbidity and the relevant outcome variable.

To evaluate the performance of the resulting predictive models, we calculated the Akaike information criterion (AIC) [29], the deviance-based R-square (R^2^) and the area under the receiver operating characteristic (AUROC) curve [30].

Statistical analyses were performed using SPSS software, version 18.0. All statistical tests and confidence intervals were constructed with a type I error (alpha) level of 5%, and *p*-values < 0.05, were considered statistically significant.

## Results

The study group included 6,102,595 cases with an average age of 49.1 ± 18.2 years, 51.3% women with a mean age of 50.3 ± 18.9 years. Main descriptive statistics for each of the outcome variables is indicated in Table 1 (see detailed information in Additional file 1: Tables S1–S6).

**Table 1:** Mean values of the six outcome variables by gender, age groups and socio-economic status

	n	PC visits (mean, ±SD, CI95%)	Frequent attenders	Home care users	Social worker users	Medications (mean, ±SD, CI95%)	Polypharmacy patients
Total	6,102,595	6.11, ±9.5, 6.10–6.12	14.1%	4.8%	2.7%	3.90 ± 4.7 3.90–3.90	15.5%
Gender							
Males	2,971,861	5.37, ±9.3, 5.36–5.38	11.8%	3.6%	1.9%	3.31, ±4.4, 3.30–3.31	12.3%
Females	3,130,734	6.82, ±9.5, 6.81–6.83	16.2%	5.9%	3.4%	4.46, ±4.9, 4.46–4.47	18.5%
Age group							
18–44 years	2,773,927	3.43, ±5.7, 3.43–3.44	5.3%	0.8%	0.7%	1.90, ±2.7, 1.90;1.90	3.3%
45–64 years	1,974,444	5.50, ±8.0, 5.49–5.51	11.8%	2.1%	1.6%	3.64, ±4.2, 3.63–3.65	12.5%
65–74 years	683,948	9.99, ±10.9, 9.96–10.01	26.9%	6.3%	3.2%	7.24, ±5.4, 7.22–7.25	35.7%
75–84 years	458,841	14.68, ±15.0, 14.64–14.72	43.2%	20.2%	10.9%	9.49, ±5.7, 9.47–9.50	53.3%
> 84 years	211,435	15.84, ±16.9, 15.77–15.91	45.4%	43.5%	18.8%	9.60, ±5.5, 9.58–9.63	55.4%
Socioeconomic status							
Very High	621,888	4.34, ±7.7, 4.33–4.36	8.7%	3.8%	2.1%	3.21, ±4.5, 3.20–3.22	12.5%
High	1,248,738	5.50, ±8.8, 4.48–5.51	12.1%	4.8%	3.0%	3.70, ±4.7, 3.69–3.71	14.6%
Moderate	2,398,649	6.39, ±9.7, 6.37–6.40	14.9%	4.8%	2.5%	3.92, ±4.7, 3.92–3.93	15.5%
Poor	1,224,004	6.82, ±10.1, 6.80–6.84	16.3%	5.4%	2.8%	4.15, ±4.8, 4.14–4.16	16.7%
Very poor	609,316	6.68, ±9.9, 6.65–6.70	15.9%	4.6%	3.4%	4.41, ±5.0, 4.40–4.43	18.3%

*PC* Primary care, *SD* Standard Deviation, *CI95%* Confidence interval 95%

**Fig. 1:**
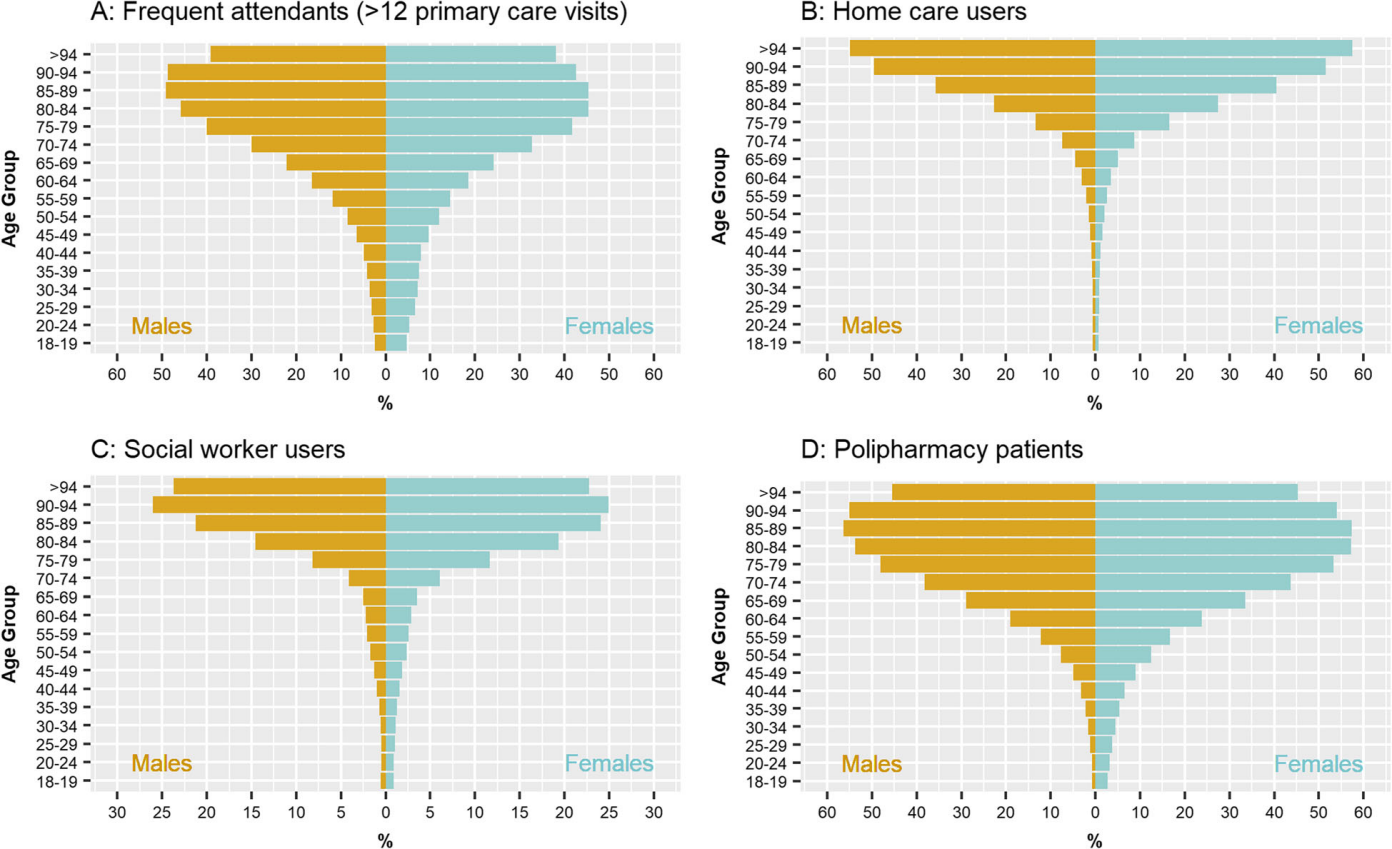
Distribution of the outcome variables in the study population by age (y-axis) and sex (x-axis)*:***a**: Frequent attendants (> 12 primary care visits) (%); **b**: Home care users (%); **c**: Social worker users (%); **d**: Polypharmacy patients (%)

Figure 1 displays the distributions of the outcome variables (A to D) by age and sex. It is of note that use of healthcare resources was slightly higher in young women than in men, but sex differences in terms of use of healthcare resources vanished above 65 years, except for use of home care services that was also higher in women above this age threshold. Table 2 describes age and sex distributions by multimorbidity measures considered in the current study.

**Table 2:** Morbidity measures by age, gender and Socioeconomic status

	n	Females (%)	Age (mean, ±SD, CI95%)	Age group (%)				Socioeconomic status (%)				
				< 65	65–74	75–84	> 84	Very High	High	Mode-rate	Poor	Very poor
Charlson index												
0	3,773,271	49.3	40.85, ± 13.8, 40.83–40.86	94.1	4.3	1.3	0.4	14.6	28.2	17.2	26.7	13.4
1	1,068,689	55.7	55.89, ± 15.8, 55.86–55.92	69.3	18.1	9.4	3.3	9.4	19.9	40.2	20.7	9.8
2	536,386	56.1	63.51, ± 14.9, 63.47–63.55	49.6	26.3	17.3	6.8	9.4	19.9	39.6	21.0	10.1
3	302,559	55.3	69.15, ± 13.7, 69.10–69.20	33.7	28.6	25.4	12.3	9.7	20.3	39.2	20.5	10.3
4	175,582	52.7	73.05, ± 12.3, 72.99;73.10	22.9	27.8	31.7	17.7	9.3	20.2	39.1	20.7	10.7
5	99,865	50.8	75.52, ± 11.5, 75.45–75.59	16.5	25.1	35.8	22.6	8.8	20.3	38.9	21.0	11.0
6	68,717	44.0	71.21, ± 16.8, 71.08–71.34	27.7	19.0	31.1	22.2	9.2	20.9	37.5	20.6	11.7
7	37,128	42.9	73.75, ± 14.6, 73.60–73.90	22.7	19.3	33.6	24.4	8.8	20.5	37.8	20.6	12.3
> 7	40,398	40.2	74.38, ± 13.5, 74.25–74.51	21.3	19.9	34.9	23.9	8.2	20.5	38.3	20.8	12.2
CRG status (^a^)												
Healthy/Non-Users	3,610,832	48.6	40.27, ± 13.5, 40.25–40.28	95.0	3.5	1.1	0.4	10.9	20.8	38.9	19.5	9.8
History of significant acute disease	111,709	55.4	47.96, ± 15.9, 47.87;48.05	83.6	10.3	4.3	1.8	8.3	19.1	40.8	21.1	10.7
Single minor chronic disease	423,418	65.8	52.35, ± 14.6, 52.31–52.39	79.2	14.1	5.0	1.6	9.3	20.2	40.6	20.4	9.5
Minor chronic disease in multiple systems	119,540	79.5	61.07, ± 12.8, 61.00–61.15	59.6	25.9	11.4	3.0	9.8	20.4	40.3	20.1	9.3
Single dominant or moderate chronic disease	1,026,770	50.8	61.25, ± 16.4, 61.22–61.29	54.8	22.7	15.4	7.0	9.2	20.1	39.7	20.8	10.1
Significant chronic disease in multiple systems	758,547	52.7	70.11, ± 13.5, 70.08–70.14	31.0	28.1	27.4	13.5	8.8	19.6	39.5	21.3	10.8
Dominant chronic disease in > 2 systems	36,668	55.8	74.92, ± 11.9, 74.80;75.04	18.7	22.5	37.4	21.4	8.7	19.1	38.8	21.8	11.6
Dominant and metastatic malignancies	3455	23.6	70.92, ± 14.0, 70.45–71.39	29.2	26.0	28.1	16.7	9.8	19.9	41.0	19.7	9.6
Catastrophic condition	11,656	27.7	47.97, ± 11.0, 47.77–48.17	92.5	5.6	1.6	0.2	11.9	22.2	32.6	19.6	13.7
GMA morbidity level (^a^)												
Healthy	1,007,734	41.0	40.68, ± 13.4, 40.66–40.71	95.1	3.4	1.1	0.5	16.2	22.9	36.0	16.9	8.0
Acute pathologies	531,707	43.2	36.93, ± 12.1, 36.90–36.97	97.5	1.8	0.5	0.2	10.3	20.1	40.4	19.8	9.4
Pregnancy and childbirth	79,064	100.0	32.60, ± 5.8, 32.56–32.64	100.0	0.0	0.0	0.0	6.6	18.0	40.9	22.1	12.3
Chronic pathologies in 1 system	1,231,874	47.1	40.87, ± 13.6, 40.84–40.89	94.4	3.9	1.2	0.4	9.7	20.2	40.1	20.2	9.7
Chronic pathologies in 2 or 3 systems	1,707,743	54.1	49.54, ± 16.5, 49.51–49.56	80.5	12.1	5.4	2.0	8.6	19.8	40.3	21.0	10.4
Chronic pathologies in > 3 systems	1,273,579	60.2	65.40, ± 16.1, 65.37–65.42	43.7	24.3	21.3	10.7	8.2	19.9	39.4	21.1	11.3
Active neoplasm	270,894	50.5	67.94, ± 14.5, 67.89–68.00	36.1	27.8	25.1	11.0	10.2	20.9	38.3	20.4	10.1

(^a^) See Tables 4S–6S, respectively, for further information

The contributions of each multimorbidity measure to explain the outcome variables is indicated in Table 3. The first row of the table indicates model fitting (AIC, R^2^ and AUC) for each outcome variable (columns) against a model containing age, sex, socioeconomic level and all first order interactions among those covariates without taking into account multimorbidity (baseline model). The corresponding values of these statistical measures when each of the four multimorbidity measures is added as a covariate are displayed in the subsequent rows, from 2nd to 4th. It is of note that lower values for AIC and higher values for both R^2^ and AUC indicate enhanced model fitting by including the corresponding multimorbidity variable. In general, the three statistics (AIC, R^2^ and AUC) used to assess model fitting showed acceptable concordance within each multimorbidity measure.

**Table 3:** Contributions of multimorbidity measurements on predictive modelling of use of healthcare resources in Primary Care

	A: Frequent attendants			B: Home care needs			C: Social worker needs			D: Polypharmacy patients		
	AIC	R^2^	AUC	AIC	R^2^	AUC	AIC	R^2^	AUC	AIC	R^2^	AUC
Baseline model	3.08	21.5%	0.771	1.38	33.4%	0.862	1.01	20.6%	0.809	3.27	29.9%	0.835
Charlson index	2.83	28.7%	0.808	1.30	37.6%	0.878	0.96	24.8%	0.841	2.78	41.4%	0.880
Number of chronic diseases	2.60	35.3%	0.840	1.27	38.9%	0.886	0.93	27.6%	0.862	2.44	49.1%	0.906
Clinical Risks Groups (CRG)	2.70	32.5%	0.830	1.30	37.5%	0.883	0.95	25.4%	0.851	**2.38**	**50.9%**	**0.912**
Adjusted Morbidity Groups (GMA)	**2.49**	**38.4%**	**0.853**	**1.25**	**40.0%**	**0.890**	**0.91**	**29.3%**	**0.872**	2.41	50.1%	0.910

The table reports the statistics indicating model fitting of the multiple regression analyses carried out to estimate each of the outcome variables (A to D). The first row describes absolute values of the three statistics: AIC: Akaike Information Criterion (in millions); R^2^: deviance-based R-squared measure; and AUC: Area Under the ROC Curve for predictive models including as covariates: age group, sex, socioeconomic status and all the first order interactions between these variables, but not multimorbidity measurements (Baseline model)

The subsequent rows correspond to the contributions of the four multimorbidity measures to model fitting for each outcome variable (A to D), namely: i) Charlson index; ii) Number of chronic diseases; iii) Clinical Risks Groups (CRG), and, iv) Adjusted Morbidity Groups (GMA)

The results displayed in Table 3 indicate that multimorbidity assessment provides a significant enhancement of predictions irrespective the measure applied. The comparisons among the three multimorbidity measures indicate that the best results were obtained with the use of GMA except for patients receiving polypharmacy, where GMA performed slightly below CRG.

## Discussion

### Main findings

To the best of our understanding, the current study has generated two relevant findings. Firstly, the results consistently confirm that inclusion of multimorbidity as a covariate generates a significant enhancement of estimations of use of healthcare resources in Primary Care.

Secondly, comparisons among the different multimorbidity measures indicate that GMA provided better discrimination and predictive power than the other multimorbidity measures, for all the outcome variables except for those patients receiving polypharmacy. For this outcome, the contribution of CRG was only slightly higher than that of GMA. It is of note, however, that GMA shows higher applicability than CRG because the use of the former does not require information on drug prescription.

We acknowledge that predictive modelling based on registry data (CHSS) showed moderate robustness since, in the best scenario, it can only explain a rather modest percentage of the overall individual variability. Consequently, the study suggests the need for exploring synergies between the GMA grading system and functional status (i.e. mobility, strength, cognitive status), as well as clinical information, to enhance health risk assessment and service selection in the clinical arena, as analysed in detail in [18].

### Contributions beyond the current state of the art

As mentioned, the current research supports previous findings indicating that assessment of multimorbidity enhances predictive modelling of use of healthcare and social support resources at community level as compared to approaches based only on demographics. It can be speculated that the modest performance of the Charlson index in the study can be partly explained by the fact that its calculation is based on a reduced number of disorders (*n* = 20) using fixed-weights in each of them and focused only in mortality.

The number of chronic diseases, despite its simplicity, shows better predictive value than the CRG for most of the outcomes, except for polypharmacy. It is of note, however, that the GMA shows the best performance.

While several studies have analysed the contribution of the Adjusted Clinical Groups (ACG) System [31] as a multimorbidity index for prediction of use of healthcare resources [3, 32], there are few analyses assessing the role of CRG as multimorbidity index. Orueta et al. [9] have reported that CRG behaves similarly to ACG for prediction of use of healthcare resources in primary care. As alluded to above, the current research indicates lower performance for CRG as compared to GMA, except for polypharmacy outcome.

### Strengths and weaknesses of the study

The clinical focus of the study assessing main outcome variables, namely: (i) Frequentation in Primary Care, (ii) Integration with social support services; and, (iii) Pharmacological prescription, should be considered a novelty and, consequently, a strength of the current research. Also, the characteristics of the source dataset in terms of quality of the CHSS registry information and extension of the dataset should be considered as key factors providing robustness to the predictive modelling.

We acknowledge, however, that assessment of social support services shows some limitations since it does not include the entire study population. Moreover, the covariate on socioeconomic level did not rely on individual information. Instead, it was based on average data of the primary care area.

We also acknowledge that GMA, as well as other multimorbidity indices, show limitations for risk assessment of specific subsets of individuals like children or patients with mental disorders. However, undergoing research on mental illnesses is opening novel perspectives in this area.

### Implications beyond the current study

The rather modest amount of inter-individual variability explained by the current predictive modelling seems to indicate that integration between registry data from the CHSS and electronic medical records of healthcare providers emerge as a high priority goal in order to enhance clinical predictions that may facilitate links between health risk predictive modelling and integrated care service selection [18]. This approach should pave the way toward enhanced risk assessment with huge positive implications on clinical management of chronic patients by assisting health professionals in clinical decision-making and in optimizing their agendas.

Besides the high potential of multimorbidity assessment in the clinical scenario, the current approach shows also interest for macro level management of chronic conditions. For example, it can be extremely useful in several areas, namely: (i) service commissioning, (ii) design of reimbursement incentives; (iii) benchmarking among providers; (iv) propensity score statistical weighting in studies carried out in a real world scenario [33], etc. But, it has also shown to be useful for linking macro and micro level management for the design of services with case finding purposes addressing patients with high risk of undesirable health events [19].

It should be highlighted that, besides the predictive performance provided by the GMA in the current study, the rationale behind its use, against alternative health risk assessment tools, is that GMA complies with four main recommended criteria [18], namely: (i) Population health approach (uses the entire population of 7.5 M inhabitants of the region); (ii) Publicly owned without licensing constraints; (iii) Based on open source computational algorithms; and, iv) Adjusted morbidity grouper that relies mostly on statistical criteria, as opposed to other tools that include expert-based coefficients, thus facilitating quick transferability to other territories.

## Conclusions

The current study confirms the impact of multimorbidity assessment for enhanced predictive modelling of use of healthcare resources in Primary Care through a population health approach. Moreover, the research indicates the high potential of GMA in terms of performance and applicability.

## Supplementary information

**Additional file 1.** Characteristics of the GMA algorithm and extended information on the study group.

## Data Availability

The datasets generated and/or analysed during the current study are not publicly available due limitations of the EU General Data Protection Regulation (GDPR) but are available from the co-author David Monterde (dmonterde@gencat.cat) on reasonable request.

## Abbreviations

ACG: Adjusted Clinical Groups
AHRQ: Agency for Healthcare Research and Quality
AIC: Akaike information criterion
AUC/AUROC: Area under the ROC Characteristic
CCI: Chronic Condition Indicator
CCS: Clinical Classifications Software
CHSS: Catalan Health Department surveillance system
CRG: Clinical Risk Groups
GMA: Adjusted Morbidity Groups
HCB: Hospital Clínico de Barcelona
HCUP: Healthcare Cost and Utilization Project
