# Supplementary material for: Multimorbidity as a predictor of health service utilization in primary care: a registry-based study of the Catalan population
Source: BMC Fam Pract. 2020 Feb 17;21:39. doi: 10.1186/s12875-020-01104-1 (PMC7026948; doi:10.1186/s12875-020-01104-1)
Supplement: Supplementary file 1 — Additional file 1. Characteristics of the GMA algorithm and extended information on the study group. [file 12875_2020_1104_MOESM1_ESM.docx]

**Multimorbidity as a predictor of health service utilization in Primary Care: a registry-based study of the Catalan population**

*Monterde D et al*

**ON-LINE SUPPLEMENTARY MATERIAL**

Section I of the current document provides a summary of the logics of the Adjusted Morbidity Grouper (GMA) algorithm (**Figure 1S**)*,* as well as *a*dditional information on methodological aspects of the GMA, described in references [18, 19]; whereas, Section II of the document displays the supplementary Tables indicated in the main document.

**Section I - Summary of the Adjusted Morbidity Grouper (GMA) algorithm**

**Basic concepts -** The input for the GMA algorithm is a text file containing information about the health problems (diagnoses) of the insured. Each record in the input file corresponds to a health problem. The required fields are:

- identification of the insured
- diagnostic classification used
- code of the health problem
- date of diagnosis
- birthdate
- sex of the insured

The fields related to date of birth and sex are not considered for grouping, but to carry out prior validation of diagnostic codes and to avoid inconsistencies.

Note that we use the date of diagnosis and not the date of the contact. In fact, in the GMA what interests us is to know the morbidity of the insured and not the complexity of the admission.

There are different classifications and versions of diagnostic coding. The GMA grouper is prepared to work with codes of the international classifications ICD-9 CM, ICD-10, ICD-10-CM, CIAP-1 and CIAP-2. The system can handle the usage of different classifications simultaneously, for example when one classification is used in primary care and another type in the specialized care. That is why the coding is needed to be identified at each case (record).

The level of specificity depends on the classification used and may exceed 90,000 codes. The GMA algorithm uses Diagnostic Code Groups (ACDs) with the intention of reducing the number of codes by associating each code with a disease (e.g. all COPD diagnosis codes are linked to one ACD group). For the grouping, a modified version of the Clinical Classification Software of the H-CUP is used [https://www.hcup-us.ahrq.gov/toolssoftware/ccs/ccs.jsp].

Chronic diseases bear with a special importance in the GMA algorithm. Therefore, this attribute is needed to be identified for all diagnostic codes. To this end, we use a modification of the Chronic Condition Indicator of the H-CUP [https://www.hcup-us.ahrq.gov/toolssoftware/chronic/chronic.jsp].

Oncological diseases and pregnancy and/or childbirth are also treated in a distinct way, and just as in the case of chronic diseases, they are identified separately. For these diagnoses, the identifications of the diagnostic codes were carried out and reviewed one-by-one for each of the four classification systems used.

Finally, at the level of diagnostic codes, it is also important to identify the affected organ system. This will be of vital importance when analysing the multimorbidity of insured persons with chronic diseases. In the case of the GMA, it is particularly important to identify the different systems affected by chronic diseases and not so much to have different chronic diseases. As in the previous cases, each diagnostic code must be identified to the corresponding organ system. To this end, we use the classification of the CIE: Infections, neoplasms, digestive system, circulatory system, etc ...

Thus, for each classification (ICD-9-CM, ICD-10, ICD-10-CM, CIAP-1 and CIAP-2), we generate a table with the previous diagnostic information associated with each diagnostic code:

- Diagnostic code
- Identification of chronicity
- Identification of acute pathology
- Identification of neoplasia
- Identification of pregnancy and/or childbirth
- Identification of the affected organ system
- Identification of Diagnostic Code Grouping (ACD)

Once the above information is obtained, ACD is used as the level of internal diagnostic analysis (minimum unit of analysis). At the level of the ACD, we assessed care needs (complexity) using the Catalan Health Information System. Specifically, to perform the initial analysis, we used the population insured by the Catalan Health Service in 2011 (7.5 million inhabitants).

Several indicators were assessed when trying to measure complexity in terms of morbidity for each ACD: mortality, hospital admissions (scheduled and unscheduled), primary care visits and prescription. To summarise, we can say that complexity is gathered from three large blocks of information indicated below, bearing in mind that all of them refer to all areas and levels of care:

- Mortality
- Health care needs
- Prescriptions

By means of statistical modelling, the previous indicators are represented as a numerical value of complexity (relative weight) for each ACD. This relative weight or complexity index for ACD will allow us to assign to each insured a level of total complexity depending on their morbidity.

Furthermore, we identify relevant ACDs. From the grouping of codes of relevant pathologies, we form clinical labels, whose objective is to summarize the set of relevant diseases present in an insured. In summary, at ACD level, we assign two fields of vital importance:

- Relative complexity weight
- Relevant pathology label

The clinical label is kept empty when the pathology is not considered relevant. Finally, it should be mentioned that the grouping is carried out for a relatively long period (usually one year), so it is necessary to set a start and end date.

**Diagnostic information -** At this point, the grouping algorithm contains the necessary tables to obtain the diagnostic information for each health problem, regardless of the classification used (ICD-9-CM, ICD-10, ICD-10-CM, CIAP-1, CIAP-2). For each diagnostic code, we have the following information:

- Diagnostic code
- Identification of chronicity
- Identification of acute pathology
- Identification of neoplasm
- Identification of pregnancy and/or childbirth
- Identification of the affected organ system
- Identification of Diagnostic Code Grouping (ACD)
- Relative weight of complexity of ACD
- Relevant pathology label

***Figure S1****. The figure shows a scheme of the grouping algorithm.*

**Treatment of diagnostic registries of the input file -** The grouper analyses each diagnostic code, registry to registry.

- **Validation of the diagnostic code** based on the age and gender of the insured is carried out. If an inconsistency is found the health problem is not taken into account by the grouper. With the inconsistent diagnostic codes a validation file is created so that it can be revised later.
- **Identification of diagnostic information** for each case based on the internal tables developed for the diagnoses (as explained above). From the code of the health problem and the classification used, we identify whether the diagnosis is chronic or acute, whether it corresponds to a neoplasm or pregnancy and/or childbirth, the organ system it affects, the ACD assigned, the relative weight of complexity and if it corresponds to a relevant pathology.
- **Verification of the diagnosis date**. Health problems diagnosed after the end or acute health problems diagnosed prior to the beginning of the analysed period are excluded.

**Treatment of registries at the insured level -** Having all the diagnostic information available (ACD, chronicity, neoplasia, organic system, ...) about all the health problems of an insured, we further process the registries following the steps below:

- **Identification of active neoplasia**: It is done, if the insured presents any ACD associated to any diagnosis of neoplasm with a diagnosis date within the analysis period or a year before.
- **Identification of pregnancy and/or childbirth**: It is done, if the insured has any ACD associated with a diagnosis of pregnancy and/or childbirth with a diagnosis date within the analysis period.
- **Identification of the acute pathology**: It is done, if the insured has any ACD associated with an acute diagnosis with a diagnosis date within the analysis period.
- **Identification and treatment of chronic pathology**: It is done, if the insured has any ACD associated with a chronic diagnosis either within the analysis period or as a previous diagnosis. In this case, both the total number of ACDs associated with chronic pathology (number of chronic diseases) and the total number of systems affected by chronic diseases are analysed.
- **Assignment of complexity by insured (relative weight)**: The grouper assigns a numerical value as a relative measure of complexity at individual level. This value is assigned for each ACD present in the insured, and not by diagnostic codes per se.
- **Assignment of relevant disease label per insured**: The grouper also assigns a summary label with the relevant diseases identified in the insured. In order to do so, the classification tables (CIE-9-CM, ICD-10, ICD-10-CM, CIAP-1 and CIAP-2) identify 80 diseases that are considered relevant, with their respective labels. Whenever one of these pathologies is identified in the same insured, the grouper saves them to form the final label. In case the relevant disease corresponds to a neoplasia in the label, it is taken into account whether it is active or prior.

**Preparation of the output file with information at the insured level** - The information at the insured level obtained at this point is as follows:

- Identification of neoplasia
- identification of disease associated with pregnancy and/or childbirth
- identification of acute disease
- number of chronic diseases
- number of organ systems affected by chronic disease
- total relative weight (complexity)
- list of relevant disease labels (if any)

From the information above, the following tasks are carried out on the insured level:

- **Assignment of the Morbidity Group (GM):** The GM assignment is carried out by priority levels according to the following sequence (only the first fulfilled condition is considered):
- If the insured is identified as ill with active neoplasia then this group of morbidity is assigned (GM = '40')
- If the insured is identified with pregnancy and/or childbirth-related pathology then this morbidity group is assigned (GM = '20')
- If the insured has 4 or more systems affected by chronic illness then this group of morbidity is assigned (GM = '33')
- If the insured has 2 or 3 systems affected by chronic disease then this group of morbidity is assigned (GM = '32')
- If the insured has 1 system affected by chronic illness then this group of morbidity is assigned (GM = '31')
- If the insured has an acute illness (Acute = 1) identified then this group of morbidity is assigned (GM = '10')
- Lastly, the healthy population morbidity group (GM = '00') is assigned
- **Assignment of the level of complexity (C):** The assignment of the level of complexity of the insured is carried out separately by groups of morbidity and taking into account the total complexity. Within each morbidity group, 4 cutoff points are identified from the 40, 70, 85, and 95 percentiles obtained from the entire population used for the creation of the cluster. The above values are fixed in the algorithm.
- **Assignment of the GMA per insured:** The GMA code consists of three digits and is assigned by combining the morbidity group (GM) and the complexity level (1 to 5): GMA = 'GM' + 'C'
- **Review and update of the insured's final clinical label:** The insured's final label is formed by concatenating the tags identified in the insured's label listing. A priority (relevance) code is used for this purpose. However, in many insured people (including all of the healthy population) there is no identified label. If there is no relevant label identified and the morbidity group is the healthy population, acute pathology or pregnancy and/or childbirth (GM = '00', GM = '10', GM = '20' respectively) then is labeled 'No chronic pathology'. If the insured has chronic illness, but is not considered relevant, he is labeled "other chronic diseases".
- **Results returned per insured.** The final fields that the grouper returns are:
- Identity of the insured
- Adjusted Morbidity Group Code
- Number of chronic diseases present
- Number of organic systems affected by chronic disease
- Total relative weight of the insured (complexity)
- Clinical label summary of the set of relevant diseases identified in the insured.

**Risk classification using GMA -** The use of the GMA grouper provides allocation of each citizen into the risk stratification pyramid. A summary representation of the update carried out by the end of 2014 grouping the results in four main risk strata is depicted in **Figure 2S**. The four main strata are identified according to the criteria indicated below:

• **GMA-1** corresponds to 50% of the population, with a lower complexity level.

• **GMA-2 or low risk stratum**: it corresponds to 30% of the population, which has higher complexity than the previous risk stratum.

• **GMA-3 or moderate risk stratum**: it corresponds to 15% of the population, which has greater complexity than the previous risk stratum.

• **GMA-4 or high-risk stratum**: it corresponds to 5% of the population, which has the highest complexity level.

***Figure S2*** *- Stratification of the Catalan population (2014) using the GMA. The third and fourth columns depict rates of mortality and hospital admissions, respectively. The fifth column indicates the cost per inhabitant per year expressed in € and the last column refers the percentage of total healthcare expenditure by risk strata. It is of note that the closer the patient is to the tip of the pyramid, the higher are: mortality, risk of hospital admission and healthcare expenses. Green color (bottom) indicates healthy status whereas red (tip) corresponds to maximum risk of admissions and highest mortality risk.*

**Clinical assessment -** A comparative clinical evaluation of GMA and CRG classifications was blindly undertaken by 40 general practitioners examining electronic heath records from 1000 cases (25 cases per general practitioner). An analysis of concordance among clinical evaluators was carried out. The analysis of the results was focused on identification of the discrepancies between the two morbidity groupers. The description of methodological aspects of the clinical validation, as well as a detailed report of the results can be found in [5]. Briefly, the results (**Figure 3S**) indicate that the two morbidity groupers (GMA and CRG) agreed with clinicians in the classification of the population by complexity, but GMA shows a better performance in the strata of greater complexity. Moreover, in most cases, clinical evaluators preferred GMA [Clèries M, et al. Validación clínica de 2 agrupadores de morbilidad en el ámbito de atención primaria. Aten Primaria. 2019. https://doi.org/10.1016/j.aprim.2018.09.016].


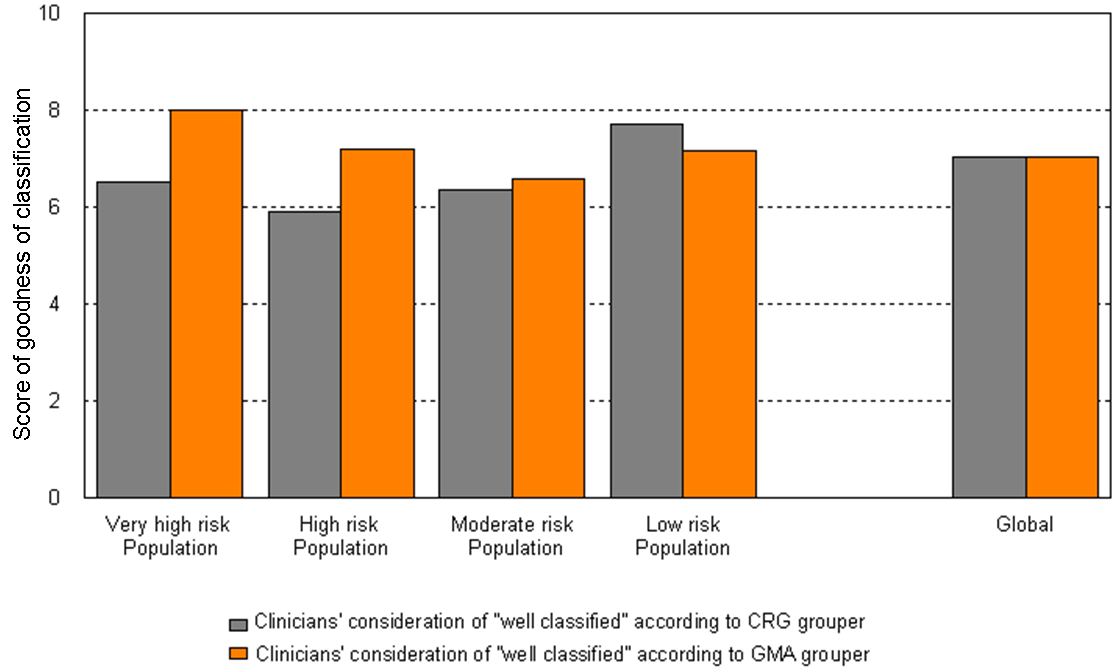


***Figure S3****: Goodness of the classifications generated by the two morbidity groupers: CRG (grey) and GMA (orange) by level of complexity assigned by the general practitioner. The last column provides a summary analysis.*

**Section II: supplementary tables**

***Table S1****: Use of Health care resoures in Primary Care by gender and by age groups*

|  | Number of population | | Primary care visits (mean) | | Frequent attenders (%) | | Home care users (%) | | Social worker users (%) | | Medications (mean) | | Polypharmacy patients (%) | |
| --- | --- | --- | --- | --- | --- | --- | --- | --- | --- | --- | --- | --- | --- | --- |
|  | Male | Females | Male | Females | Male | Females | Male | Females | Male | Females | Male | Females | Male | Females |
| 18-19 | 70,460 | 65,216 | 2.4 | 3.6 | 2.4 | 4.7 | 0.5 | 0.7 | 0.6 | 0.9 | 1.2 | 2.0 | 0.7 | 2.7 |
| 20-24 | 180,924 | 172,630 | 2.5 | 3.7 | 2.7 | 5.2 | 0.6 | 0.7 | 0.5 | 0.9 | 1.2 | 2.1 | 0.8 | 3.1 |
| 25-29 | 205,182 | 208,750 | 2.6 | 3.9 | 3.1 | 6.6 | 0.6 | 0.8 | 0.5 | 1.0 | 1.3 | 2.2 | 1.1 | 3.8 |
| 30-34 | 272,673 | 270,588 | 2.7 | 4.1 | 3.5 | 7.1 | 0.6 | 0.9 | 0.6 | 1.2 | 1.4 | 2.3 | 1.6 | 4.5 |
| 35-39 | 348,258 | 327,962 | 2.9 | 4.2 | 4.1 | 7.4 | 0.7 | 1.1 | 0.7 | 1.3 | 1.6 | 2.4 | 2.2 | 5.3 |
| 40-44 | 337,342 | 313,942 | 3.2 | 4.3 | 4.9 | 7.8 | 0.9 | 1.2 | 1.0 | 1.5 | 1.8 | 2.6 | 3.2 | 6.6 |
| 45-49 | 299,176 | 286,290 | 3.7 | 4.8 | 6.4 | 9.6 | 1.2 | 1.5 | 1.3 | 1.8 | 2.2 | 3.1 | 5.0 | 9.0 |
| 50-54 | 261,563 | 262,841 | 4.5 | 5.6 | 8.5 | 12.0 | 1.5 | 2.0 | 1.7 | 2.3 | 2.8 | 3.7 | 7.7 | 12.4 |
| 55-59 | 226,631 | 237,389 | 5.6 | 6.3 | 11.8 | 14.4 | 2.1 | 2.6 | 2.0 | 2.6 | 3.6 | 4.4 | 12.2 | 16.7 |
| 60-64 | 192,748 | 207,806 | 7.1 | 7.6 | 16.6 | 18.5 | 3.0 | 3.5 | 2.2 | 2.8 | 4.9 | 5.6 | 19.1 | 23.9 |
| 65-69 | 178,360 | 199,695 | 8.9 | 9.2 | 22.1 | 24.1 | 4.5 | 5.1 | 2.5 | 3.5 | 6.4 | 6.9 | 28.9 | 33.6 |
| 70-74 | 140,909 | 164,984 | 10.9 | 11.4 | 30.0 | 32.7 | 7.4 | 8.7 | 4.1 | 6.1 | 7.6 | 8.3 | 38.3 | 43.6 |
| 75-79 | 102,413 | 134,247 | 13.7 | 14.0 | 40.1 | 41.7 | 13.4 | 16.5 | 8.2 | 11.6 | 8.9 | 9.5 | 48.1 | 53.2 |
| 80-84 | 87,930 | 134,251 | 15.8 | 15.4 | 45.9 | 45.4 | 22.6 | 27.4 | 14.5 | 19.4 | 9.5 | 9.9 | 53.7 | 57.3 |
| 85-89 | 47,242 | 89,972 | 17.1 | 15.8 | 49.1 | 45.4 | 35.7 | 40.4 | 21.3 | 24.0 | 9.8 | 9.9 | 56.3 | 57.3 |
| 90-94 | 16,906 | 42,404 | 16.9 | 15.0 | 48.7 | 42.6 | 49.6 | 51.5 | 26.0 | 25.0 | 9.5 | 9.3 | 55.1 | 54.0 |
| >94 | 3,144 | 11,767 | 13.9 | 13.5 | 39.1 | 38.1 | 54.9 | 57.5 | 23.7 | 22.8 | 8.1 | 8.2 | 45.5 | 45.3 |
| Total | 2,971,861 | 3,130,734 | 5.4 | 6.8 | 11.7 | 16.3 | 3.6 | 5.9 | 1.9 | 3.4 | 3.3 | 4.5 | 12.3 | 18.5 |

***Table S2****: Number of co-morbid disorders expressed as number of body systems affected by gender and age groups*

|  | 0 | | 1 | | 2-3 | | >3 | |
| --- | --- | --- | --- | --- | --- | --- | --- | --- |
|  | Male | Females | Male | Females | Male | Females | Male | Females |
| 18-19 | 45.1 | 39.7 | 31.0 | 29.1 | 21.5 | 26.4 | 2.4 | 4.7 |
| 20-24 | 47.6 | 37.0 | 30.9 | 29.5 | 19.4 | 28.0 | 2.1 | 5.5 |
| 25-29 | 49.3 | 35.5 | 30.6 | 29.6 | 18.2 | 28.9 | 2.0 | 6.0 |
| 30-34 | 47.0 | 32.9 | 30.7 | 29.4 | 19.9 | 30.5 | 2.5 | 7.2 |
| 35-39 | 42.8 | 30.1 | 30.3 | 28.7 | 23.2 | 32.2 | 3.7 | 8.9 |
| 40-44 | 38.7 | 28.5 | 28.3 | 26.9 | 26.9 | 33.3 | 6.1 | 11.3 |
| 45-49 | 32.9 | 25.0 | 25.5 | 23.5 | 31.3 | 35.3 | 10.3 | 16.2 |
| 50-54 | 26.5 | 20.5 | 21.8 | 19.3 | 35.1 | 36.9 | 16.6 | 23.4 |
| 55-59 | 20.5 | 15.7 | 17.6 | 15.6 | 36.9 | 37.8 | 25.0 | 30.8 |
| 60-64 | 13.8 | 10.8 | 13.0 | 12.1 | 36.6 | 37.5 | 36.5 | 39.6 |
| 65-69 | 8.1 | 7.1 | 8.6 | 8.4 | 33.9 | 34.7 | 49.5 | 49.8 |
| 70-74 | 5.1 | 4.8 | 5.9 | 5.6 | 28.9 | 29.6 | 60.1 | 60.0 |
| 75-79 | 3.2 | 3.2 | 3.9 | 3.7 | 22.9 | 23.4 | 70.0 | 69.7 |
| 80-84 | 2.5 | 2.6 | 3.0 | 2.8 | 19.0 | 19.3 | 75.6 | 75.3 |
| 85-89 | 2.3 | 2.4 | 2.4 | 2.4 | 16.5 | 16.9 | 78.8 | 78.3 |
| 90-94 | 3.1 | 2.8 | 2.5 | 2.5 | 16.7 | 17.0 | 77.7 | 77.6 |
| >94 | 9.9 | 5.8 | 3.4 | 3.1 | 18.6 | 19.6 | 68.0 | 71.4 |
| Total | 30.2 | 21.1 | 22.0 | 19.4 | 27.1 | 31.4 | 20.7 | 28.0 |

***Table S3****: GMA risk strata by gender and age groups*

|  | Baseline | | Low | | Moderate | | High | | Very high | |
| --- | --- | --- | --- | --- | --- | --- | --- | --- | --- | --- |
|  | Male | Females | Male | Females | Male | Females | Male | Females | Male | Females |
| 18-19 | 81.5 | 73.6 | 17.0 | 23.4 | 1.4 | 2.8 | 0.1 | 0.2 | 0.0 | 0.0 |
| 20-24 | 80.7 | 70.5 | 17.5 | 26.1 | 1.7 | 3.2 | 0.1 | 0.2 | 0.0 | 0.0 |
| 25-29 | 79.9 | 69.1 | 18.0 | 27.1 | 2.0 | 3.6 | 0.2 | 0.2 | 0.0 | 0.0 |
| 30-34 | 78.2 | 67.3 | 19.2 | 28.2 | 2.3 | 4.2 | 0.2 | 0.3 | 0.0 | 0.0 |
| 35-39 | 74.8 | 65.2 | 21.7 | 29.5 | 3.1 | 4.9 | 0.3 | 0.4 | 0.0 | 0.1 |
| 40-44 | 70.2 | 62.9 | 24.8 | 30.4 | 4.4 | 6.0 | 0.5 | 0.6 | 0.1 | 0.1 |
| 45-49 | 62.8 | 56.4 | 29.1 | 34.0 | 6.9 | 8.5 | 0.9 | 1.0 | 0.2 | 0.2 |
| 50-54 | 53.3 | 47.6 | 34.0 | 38.4 | 10.7 | 12.3 | 1.7 | 1.5 | 0.3 | 0.2 |
| 55-59 | 42.8 | 38.2 | 37.7 | 42.5 | 15.8 | 16.8 | 3.0 | 2.2 | 0.6 | 0.4 |
| 60-64 | 30.1 | 27.8 | 40.1 | 44.6 | 23.5 | 23.6 | 5.1 | 3.4 | 1.2 | 0.5 |
| 65-69 | 18.6 | 18.8 | 38.7 | 43.4 | 32.5 | 31.5 | 8.2 | 5.4 | 2.0 | 0.9 |
| 70-74 | 11.8 | 12.3 | 33.5 | 38.1 | 39.1 | 39.0 | 12.3 | 8.9 | 3.2 | 1.7 |
| 75-79 | 7.4 | 7.8 | 25.8 | 29.9 | 43.1 | 44.7 | 18.2 | 14.3 | 5.5 | 3.3 |
| 80-84 | 5.3 | 5.8 | 20.4 | 24.1 | 43.8 | 46.0 | 22.9 | 19.1 | 7.7 | 5.0 |
| 85-89 | 4.4 | 5.0 | 16.5 | 20.0 | 42.9 | 45.9 | 26.7 | 22.6 | 9.5 | 6.5 |
| 90-94 | 5.1 | 5.2 | 15.7 | 19.1 | 41.8 | 45.2 | 27.4 | 23.7 | 10.1 | 6.7 |
| >94 | 12.3 | 8.7 | 17.0 | 21.3 | 38.6 | 44.0 | 24.0 | 20.8 | 8.2 | 5.3 |
| Toral | 54.7 | 45.6 | 27.0 | 32.8 | 13.3 | 16.7 | 3.9 | 4.0 | 1.1 | 0.9 |

The thresholds defining these four subsets of individuals correspond to the percentiles 50, 80, 95 and 99 of the GMA grading for the general population.

**Table S4**: Charlson by outcomes.

| **Charlson index** | n | A) Frequent attenders PC (%) | B) Patients receiving home care support (%) | C) Patients receiving social support (%) | D) Patients receiving polypharmacy (%) |
| --- | --- | --- | --- | --- | --- |
| 0 | 3,773,271 | 4.9 | 1.0 | 0.9 | 3.3 |
| 1 | 1,068,689 | 17.6 | 4.3 | 3.4 | 18.2 |
| 2 | 536,386 | 29.1 | 8.6 | 6.0 | 36.0 |
| 3 | 302,559 | 37.7 | 14.4 | 9.6 | 48.0 |
| 4 | 175,582 | 46.2 | 21.1 | 13.4 | 61.6 |
| 5 | 99,865 | 53.1 | 28.6 | 17.9 | 71.4 |
| 6 | 68,717 | 50.0 | 29.5 | 18.3 | 66.8 |
| 7 | 37,128 | 55.7 | 35.8 | 22.4 | 75.4 |
| >7 | 40,398 | 60.1 | 44.2 | 28.2 | 81.8 |

**Table S5**: CRG by outcomes.

| **CRG status** | n | A) Frequent attenders PC (%) | B) Patients receiving home care support (%) | C) Patients receiving social support (%) | D) Patients receiving polypharmacy (%) |
| --- | --- | --- | --- | --- | --- |
| **Healthy/Non-Users** | **3,610,832** | **4.1** | **0.9** | **0.8** | **1.7** |
| Severity level 1 | 2,240,894 | 5.6 | 1.2 | 1.1 | 2.3 |
| Severity level 2 | 1,288,071 | 1.0 | 0.4 | 0.1 | 0.3 |
| Severity level 3 | 81,867 | 11.6 | 2.2 | 2.3 | 7.4 |
| **History of significant acute disease** | **111,709** | **15.6** | **3.1** | **2.6** | **11.2** |
| Severity level 1 | 21,769 | 10.6 | 1.6 | 1.2 | 7.3 |
| Severity level 2 | 89,940 | 16.8 | 3.5 | 2.9 | 12.2 |
| **Single minor chronic disease** | **423,418** | **14.1** | **3.0** | **2.6** | **11.8** |
| Severity level 1 | 298,831 | 11.8 | 2.3 | 2.0 | 8.4 |
| Severity level 2 | 124,587 | 19.7 | 4.8 | 4.1 | 20.1 |
| **Minor chronic disease in multiple systems** | **119,540** | **24.3** | **6.0** | **4.9** | **33.9** |
| Severity level 1 | 35,397 | 17.2 | 3.6 | 2.8 | 18.5 |
| Severity level 2 | 1,222 | 22.7 | 3.8 | 4.1 | 34.1 |
| Severity level 3 | 55,790 | 25.4 | 6.3 | 5.1 | 35.1 |
| Severity level 4 | 27,131 | 31.5 | 8.5 | 7.3 | 51.3 |
| **Single dominant or moderate chronic disease** | **1,026,770** | **23.7** | **7.6** | **5.3** | **25.5** |
| Severity level 1 | 836,754 | 22.0 | 7.1 | 4.9 | 22.5 |
| Severity level 2 | 121,924 | 31.2 | 8.1 | 5.9 | 35.4 |
| Severity level 3 | 62,499 | 30.1 | 12.9 | 9.1 | 44.3 |
| Severity level 4 | 1,073 | 63.5 | 23.0 | 13.2 | 60.6 |
| Severity level 5 | 4,359 | 34.3 | 12.0 | 8.6 | 50.9 |
| Severity level 6 | 161 | 29.2 | 12.4 | 15.4 | 83.9 |
| **Significant chronic disease in multiple syst.** | **758,547** | **44.3** | **18.7** | **12.2** | **63.6** |
| Severity level 1 | 414,944 | 38.3 | 14.4 | 9.5 | 53.4 |
| Severity level 2 | 164,439 | 48.4 | 20.3 | 13.4 | 70.5 |
| Severity level 3 | 124,745 | 51.1 | 25.2 | 16.0 | 77.7 |
| Severity level 4 | 47,390 | 61.5 | 31.2 | 19.3 | 87.0 |
| Severity level 5 | 6,745 | 65.7 | 34.9 | 22.2 | 93.0 |
| Severity level 6 | 284 | 54.6 | 25.0 | 17.4 | 90.5 |
| **Dominant chronic disease in >2 systems** | **36,668** | **57.7** | **36.1** | **25.4** | **85.7** |
| Severity level 1 | 19412 | 54.2 | 31.2 | 22.9 | 82.4 |
| Severity level 2 | 7805 | 56.0 | 37.5 | 26.4 | 85.3 |
| Severity level 3 | 9043 | 66.7 | 44.8 | 29.7 | 93.0 |
| Severity level 4 | 342 | 61.4 | 44.4 | 32.2 | 94.7 |
| Severity level 5 | 64 | 60.9 | 53.1 | 27.5 | 93.7 |
| Severity level 6 | 2 | 100.0 | 50.0 | 0.0 | 100.0 |
| **Dominant and metastatic malignancies** | **3,455** | **41.5** | **27.8** | **15.1** | **60.2** |
| Severity level 1 | 867 | 42.4 | 22.8 | 12.4 | 49.7 |
| Severity level 2 | 1,229 | 40.6 | 26.4 | 14.5 | 56.7 |
| Severity level 3 | 1,005 | 37.9 | 33.1 | 16.5 | 65.2 |
| Severity level 4 | 338 | 52.1 | 29.3 | 19.0 | 83.7 |
| Severity level 5 | 16 | 62.5 | 37.5 | 27.3 | 93.7 |
| **Catastrophic condition** | **11,656** | **16.9** | **6.2** | **6.9** | **38.7** |
| Severity level 1 | 329 | 31.9 | 25.5 | 17.5 | 26.1 |
| Severity level 2 | 8,132 | 13.4 | 4.9 | 5.3 | 31.4 |
| Severity level 3 | 1,640 | 30.1 | 9.0 | 11.6 | 69.1 |
| Severity level 4 | 1,329 | 18.1 | 5.6 | 7.9 | 45.6 |
| Severity level 5 | 226 | 14.6 | 5.3 | 10.0 | 56.2 |

**Table S6**: GMA by outcomes.

| **GMA** | n | A) Frequent attenders PC (%) | B) Patients receiving home care support (%) | C) Patients receiving social support (%) | D) Patients receiving polypharmacy (%) |
| --- | --- | --- | --- | --- | --- |
| **Healthy** | 1,007,734 | **0.8** | **0.4** | **0.1** | **0.5** |
| **Acute pathologies** | 531,707 | **3.0** | **0.8** | **0.5** | **1.3** |
| Complexity level 1 | 208,968 | 1.9 | 0.6 | 0.2 | 0.7 |
| Complexity level 2 | 144,740 | 2.5 | 0.8 | 0.5 | 1.1 |
| Complexity level 3 | 84,786 | 3.3 | 0.8 | 0.6 | 1.4 |
| Complexity level 4 | 56,540 | 4.5 | 0.9 | 0.8 | 2.0 |
| Complexity level 5 | 36,673 | 7.8 | 1.2 | 1.1 | 3.6 |
| **Pregnancy and childbirth** | 79,064 | **9.7** | **1.2** | **2.6** | **5.7** |
| Complexity level 1 | 17,085 | 3.4 | 0.7 | 0.8 | 1.2 |
| Complexity level 2 | 20,454 | 3.8 | 0.9 | 1.4 | 2.6 |
| Complexity level 3 | 15,063 | 8.1 | 1.0 | 2.1 | 4.4 |
| Complexity level 4 | 15,245 | 13.0 | 1.2 | 3.7 | 7.5 |
| Complexity level 5 | 11,217 | 24.0 | 2.7 | 6.9 | 17.4 |
| **Chronic pathologies in 1 system** | 1,231,874 | **4.5** | **1.0** | **0.8** | **2.4** |
| Complexity level 1 | 334,273 | 1.6 | 0.5 | 0.3 | 0.7 |
| Complexity level 2 | 339,601 | 2.8 | 0.9 | 0.5 | 1.5 |
| Complexity level 3 | 225,627 | 4.3 | 1.1 | 0.8 | 2.3 |
| Complexity level 4 | 194,042 | 6.6 | 1.4 | 1.2 | 3.5 |
| Complexity level 5 | 138,331 | 12.8 | 2.2 | 2.5 | 7.4 |
| **Chronic pathologies in 2 or 3 systems** | 1,707,743 | **12.2** | **2.8** | **2.2** | **10.8** |
| Complexity level 1 | 438,123 | 3.9 | 1.0 | 0.6 | 2.5 |
| Complexity level 2 | 503,563 | 7.6 | 1.8 | 1.2 | 5.9 |
| Complexity level 3 | 326,616 | 12.8 | 2.8 | 2.2 | 11.0 |
| Complexity level 4 | 267,450 | 19.7 | 4.3 | 3.7 | 18.4 |
| Complexity level 5 | 171,991 | 33.6 | 8.4 | 7.1 | 34.1 |
| **Chronic pathologies in >3 systems** | 1,273,579 | **36.7** | **13.8** | **9.6** | **46.6** |
| Complexity level 1 | 382,963 | 15.8 | 3.7 | 2.6 | 18.8 |
| Complexity level 2 | 437,890 | 33.7 | 9.7 | 6.9 | 43.4 |
| Complexity level 3 | 198,854 | 49.7 | 17.3 | 12.0 | 64.4 |
| Complexity level 4 | 161,674 | 60.2 | 26.9 | 18.0 | 77.1 |
| Complexity level 5 | 92,198 | 68.6 | 44.9 | 30.3 | 85.5 |
| **Active neoplasm** | 270,894 | **35.5** | **16.7** | **10.6** | **45.0** |
| Complexity level 1 | 99,841 | 15.0 | 5.0 | 3.2 | 17.1 |
| Complexity level 2 | 83,234 | 37.1 | 13.9 | 8.9 | 47.7 |
| Complexity level 3 | 41,946 | 52.5 | 24.0 | 15.3 | 68.2 |
| Complexity level 4 | 26,174 | 59.8 | 34.2 | 21.3 | 78.0 |
| Complexity level 5 | 19,699 | 63.9 | 48.4 | 31.4 | 81.6 |
